# Supplementary material for: Multi-omics characterization of the monkeypox virus infection
Source: Nat Commun. 2024 Aug 8;15:6778. doi: 10.1038/s41467-024-51074-6 (PMC11310467; doi:10.1038/s41467-024-51074-6)

Supplementary Figure 1

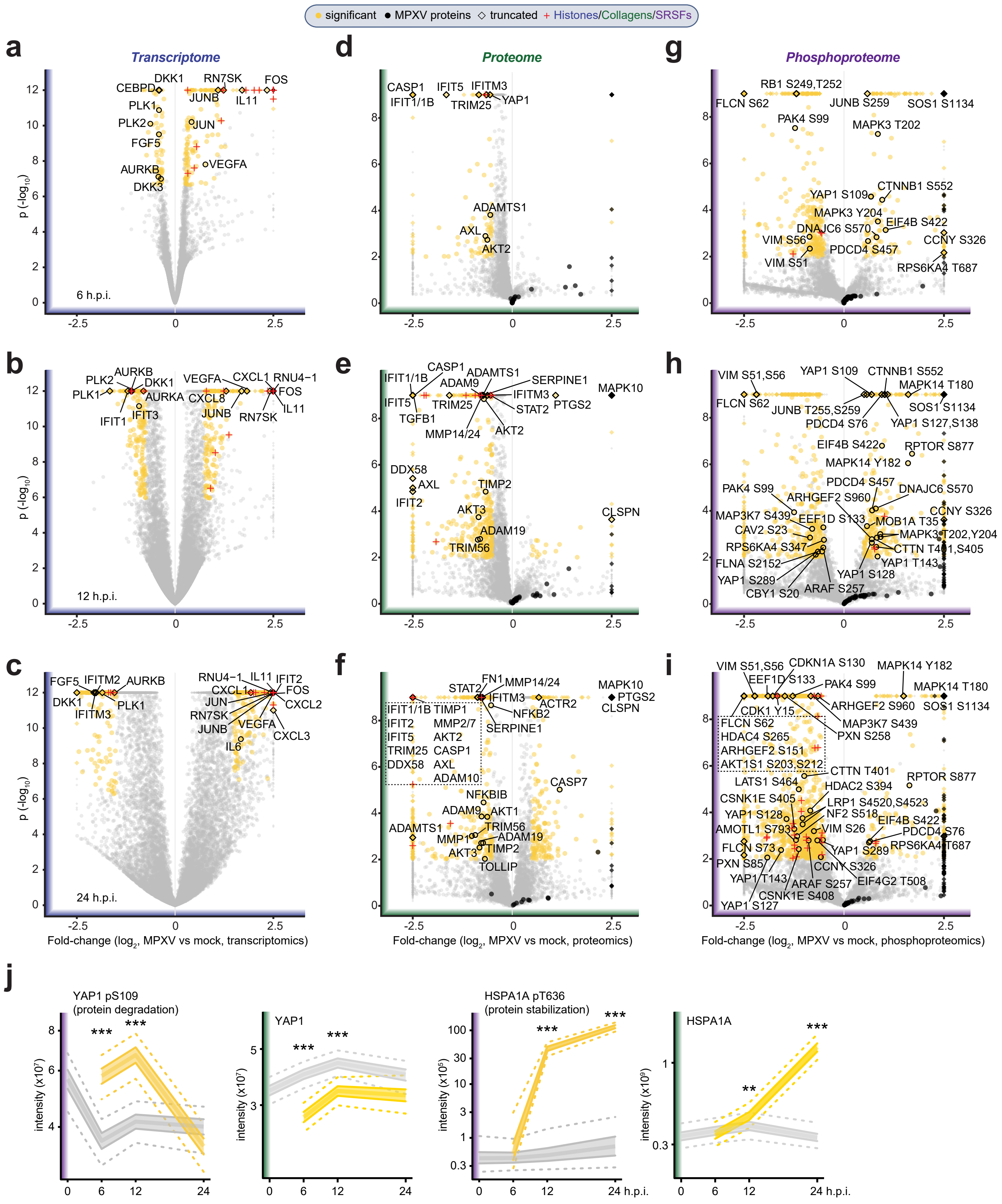

Supplementary figure 1 | MPXV infection-elicited perturbations of the host transcriptome, proteome, and phosphoproteome. (a-i) Related to Fig. 2 a-g. Primary HFFs were infected with MPXV at MOI 3 for 6, 12, or 24 hours and the RNA or protein content of infected cells were harvested for measurement of infection-induced changes on the levels of transcriptomes, proteomes and phosphoproteomes. This figure is related to the **(a-c)** Volcano plots depicting infection-induced changes to the cellular transcriptome **(a-c)**, proteome **(d-f),** or phosphoproteome **(g-i)** as indicated. Statistically significant events (see materials and methods) are highlighted in yellow, and viral transcripts, proteins, or phosphosites in black. Histones **(a-c)**, collagens **(d-f)** or Serine and arginine Rich Splicing Factors (SRSFs) **(g-i)** are further marked with crosses. Diamonds indicate that the actual log2 fold change was truncated to fit into the plot. **(j)** Modeled abundances of YAP1 S109 and HSPA1A (HSP70) T636 phosphosites as determined by phosphoproteomics analysis, depicted alongside parent protein abundances as determined by proteomics analysis of MPXV infected HFFs. These phosphorylation events were previously reported to cause protein degradation or stabilization, respectively. The line indicated the modeled median, and the shaded region and dotted line represented 50% and 95% credible intervals, respectively. Bayesian linear model-based unadjusted two-sided P-value: *: p-value ≤ 0.05; **: p-value ≤ 0.01; ***: p-value ≤ 0.001 (n = 5 independent experiments). Source data are provided as a Source Data file.

Supplementary Figure 2

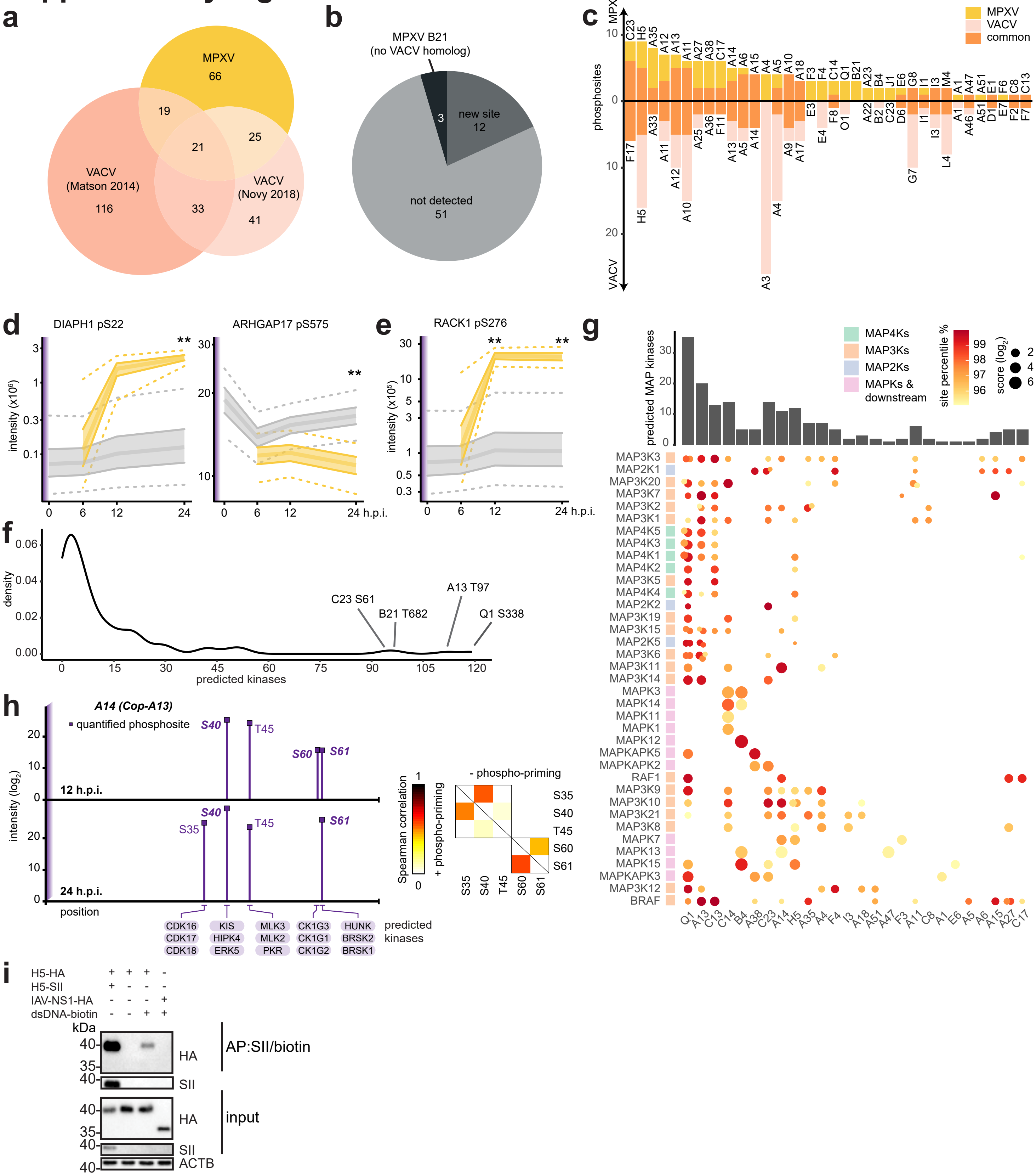

Supplementary figure 2 | Examples of MPXV infection-elicited phosphorylation changes. **(a)** Overlapping and unique phosphosites detected on MPXV from this study and VACV from previous studies[27,55]. **(b)** Additional information of the detected phosphosites on MPXV from this study but not in the VACV studies[27,55]. **(c)** Number of unique and common phosphosites identified on individual MPXV and VACV proteins. **(d)** Modeled abundances of DIAPH1 (mDia) S22 and ARHGAP17 S575 phosphosites as determined by phosphoproteomics analysis of MPXV-infected HFFs. **(e)** Modeled abundance of RACK1 S276 phosphosite as determined by phosphoproteomics analysis of MPXV infected HFFs. This site was previously shown to be phosphorylated by the Vaccinia virus kinase B1 (MPXV-B3)[58]. For **(d-e)**, the line indicated the modeled median, the shaded region and the dotted line represented 50% and 95% credible intervals, respectively. Bayesian linear model-based unadjusted two-sided P-value: *: ≤ 0.05; **: ≤ 0.01 (n = 5 independent experiments). **(f)** Related to Fig. 2e. Density plot of the number of predicted kinase motifs for individual viral phosphosites. **(g)** Related to Fig. 2e. Viral proteins harboring phosphomotifs that may be phosphorylated by MAP kinases. Each dot represents one phosphosite, and the bar plot shows the total number of predicted MAP kinases that may phosphorylate the detected phosphosites on a certain viral protein. **(h)** Related to Fig. 2d. Kinetics of phosphosites detected on the viral protein A14 (Cop-A13) along the top host kinases, recognition motifs of which were found at the respective sites. Sites that have also been identified in VACV phosphoproteome studies[27,55] are in bold italics. Spearman rank correlations between host kinases with recognition motifs at individual phosphosites (with or without phospho-priming) are further depicted (right) as a measure of the phosphosite similarity. **(i)** HEK293T cells were transfected with plasmids encoding SII-H5 and HA-H5, HA-H5 alone or HA-Nonstructural protein 1 (NS1) of influenza A virus (strain PR/8), as indicated. The lysates were used for affinity purification with Streptactin beads. Where indicated, biotin-dsDNA was spiked into the lysate. (n = 3 independent experiments, uncropped scans at the end of the document). SII: StrepII. Source data are provided as a Source Data file.

Supplementary Figure 3

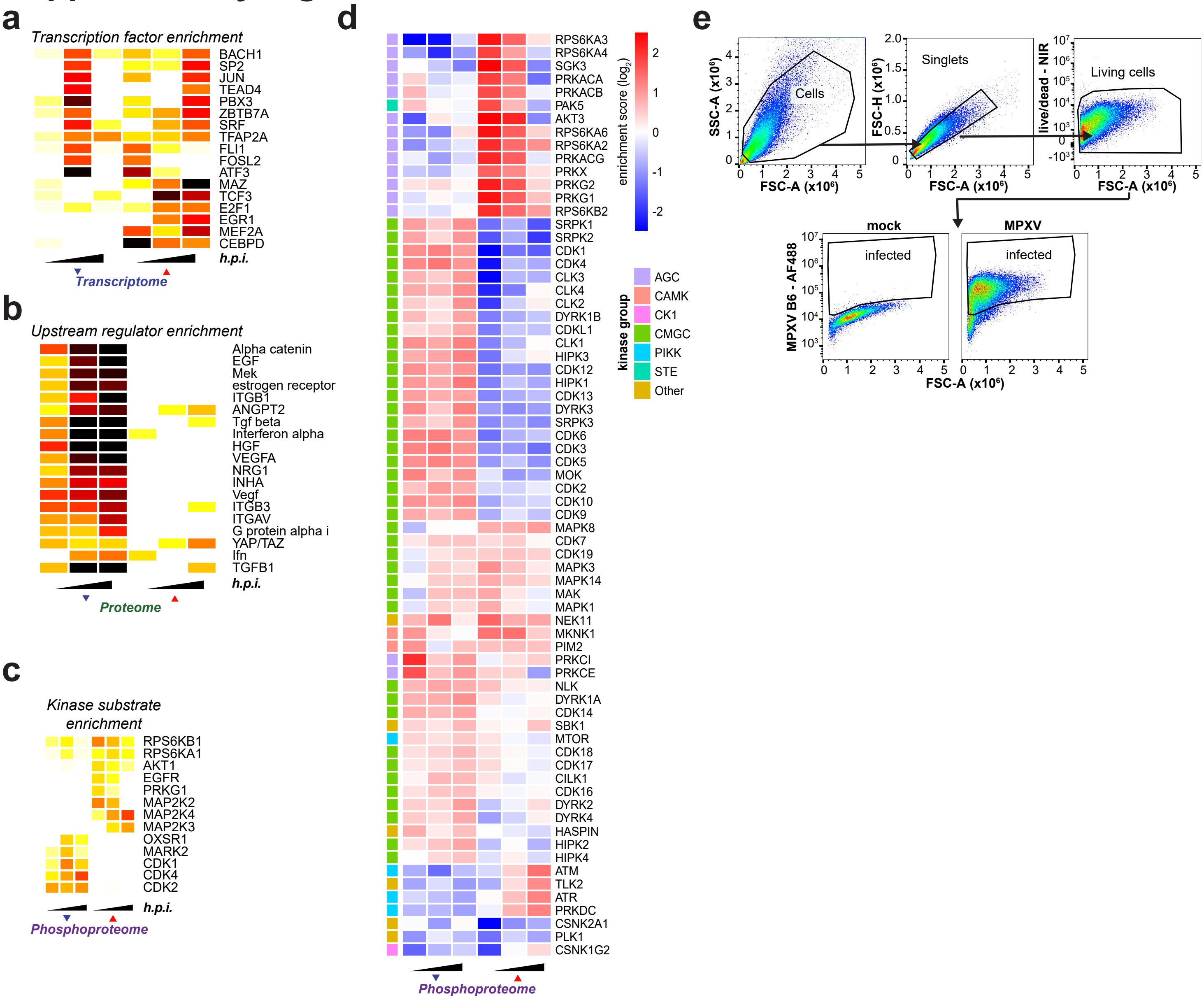

Supplementary figure 3 | Detailed results of the multi-omics systems analysis (a) The significant hits from the transcriptome analysis were used for transcription factor-target gene association (transcription factor enrichment analysis)[107,108]. Significant transcription factors (Fisher's exact test, FDR-adjusted $p < 0.001$) are depicted. **(b)** The significant hits from the proteome analysis were used for upstream regulator analysis (Ingenuity pathway analysis, Qiagen). Significant regulators (Fisher's exact test, unadjusted $p < 0.05$) belonging to the molecule types *growth factor*, *transmembrane receptor* or *group* are depicted. **(c)** The significant hits from the phosphoproteome analysis were used for kinase-substrate enrichment analysis using known kinase-substrate annotations from PhosphoSitePlus[51]. Significant kinases (Fisher's exact test, unadjusted $p \leq 0.001$) are depicted. **(d)** We performed host kinase motif enrichment analysis of significantly up- or down-regulated phosphosites from phosphoproteomics analysis of MPXV-infected HFFs based on experimentally determined substrate specificities of human S/T kinome[84] (see materials and methods). Significant kinases (log2 enrichment score > 0, Fisher's exact test FDR-adj. $p \leq 0.01$ in any condition) are depicted. **(e)** Related to Fig. 3d-f: gating strategy for the flow cytometry analysis of MPXV-affected proteins in infected HFF cells. Fig. 3d corresponds to the gating of "infected" population, and the median fluorescence intensities of the respective targets in 3e and 3f were derived from the "Living cells" population. Source data are provided as a Source Data file.

Supplementary Figure 4

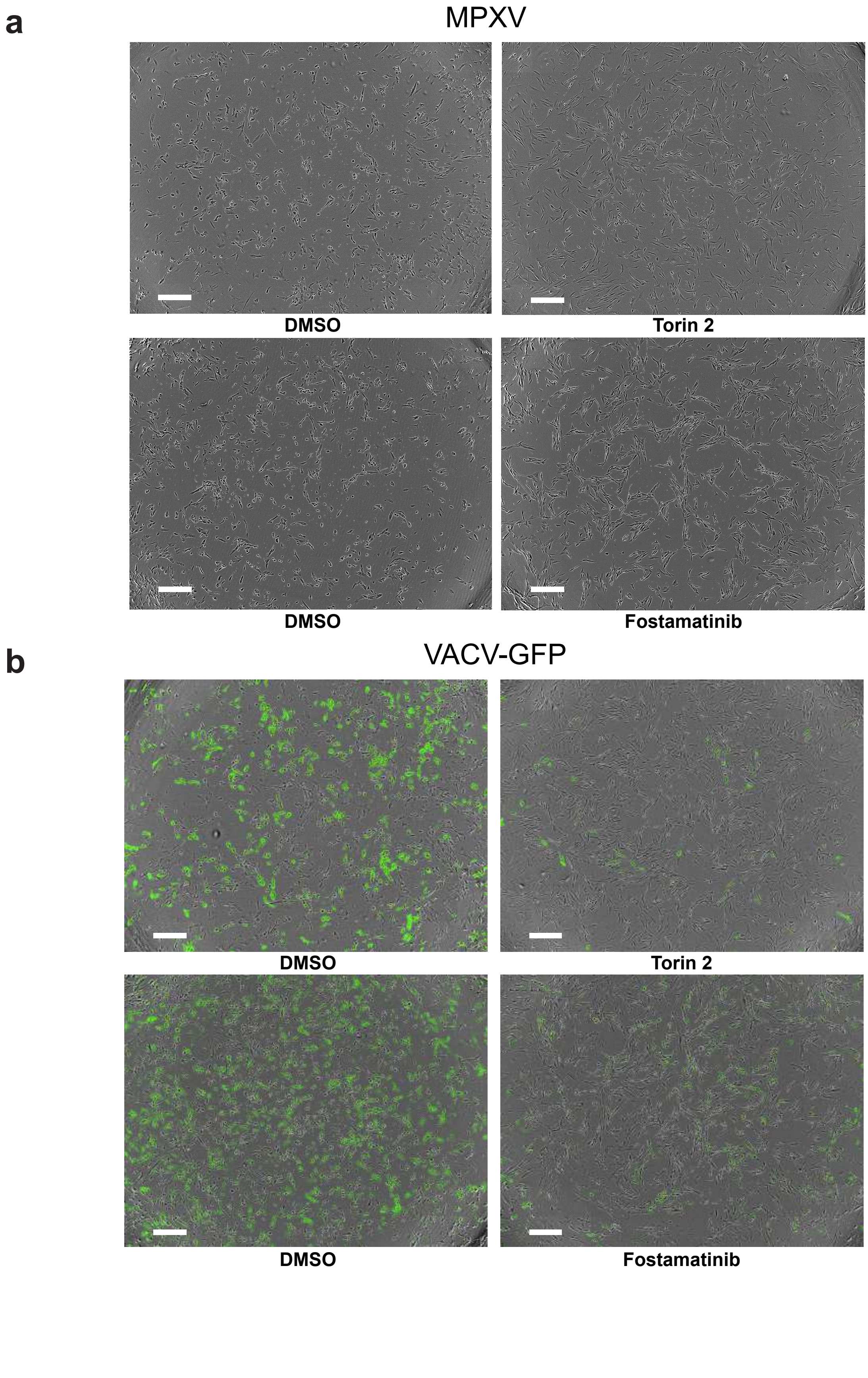

Supplementary figure 4 | Antiviral efficacy of Torin 2 and Fostamatinib upon MPXV and VACV-GFP infection. (a,b) hTERT-HFF cells were pre-treated with either DMSO, Torin 2 (1 µM), or Fostamatinib (5 µM) 4 hours before infection by MPXV **(a)** or VACV-GFP **(b)**. Representative pictures were obtained 24 hours post-infection (related to Fig. 5b, c, n = 3 independent experiments). Scale bar = 400 µm.

Supplementary Figure 2i, original files

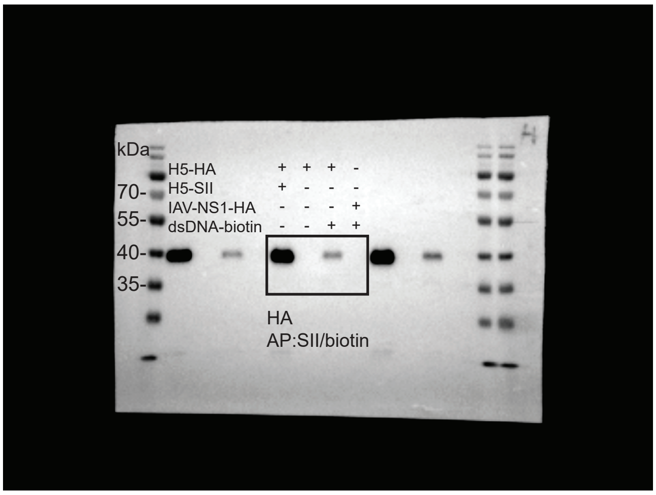

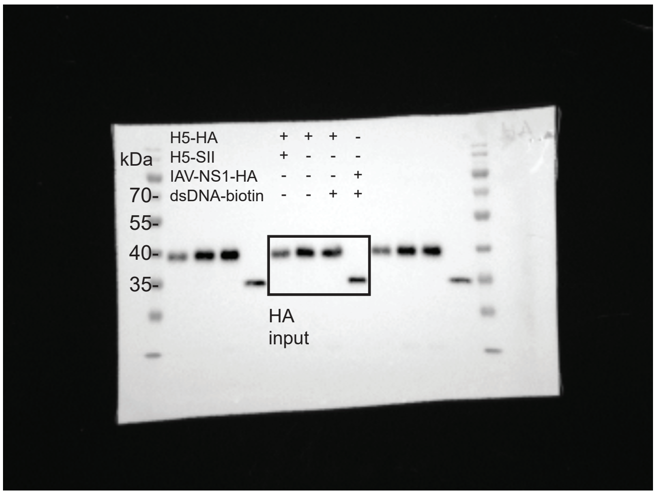

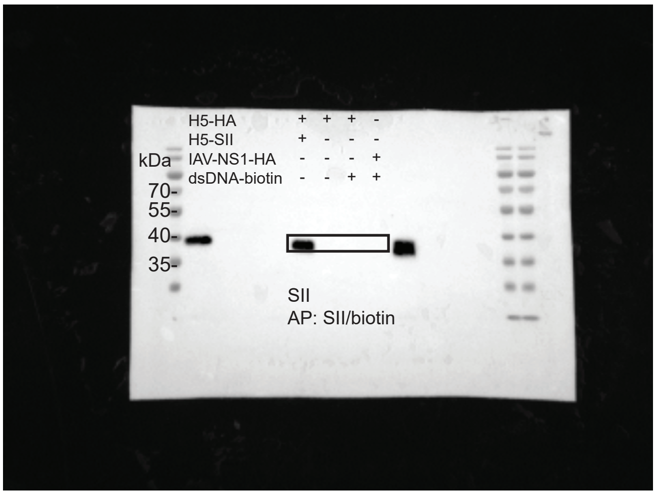

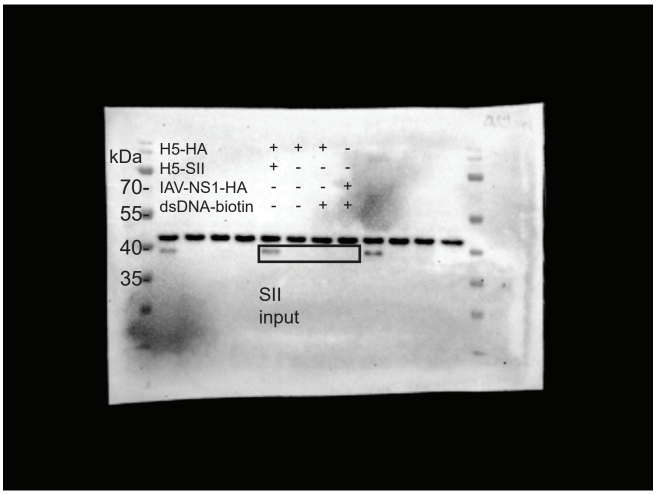

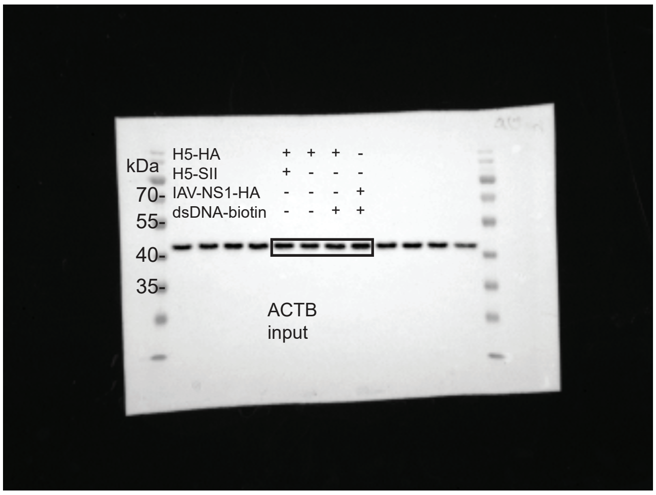

Supplement: Supplementary file 1 — Supplementary Information [file 41467_2024_51074_MOESM1_ESM.pdf]
