# Supplementary material for: Multi-omics characterization of the monkeypox virus infection
Source: Nat Commun. 2024 Aug 8;15:6778. doi: 10.1038/s41467-024-51074-6 (PMC11310467; doi:10.1038/s41467-024-51074-6)
Supplement: Supplementary file 3 — Description of Additional Supplementary Information [file 41467_2024_51074_MOESM3_ESM.docx]

Description of additional supplementary files

Title: Supplementary data 1: Protein sequence alignment between MPXV and VACV

Description: Portein sequence alignment between the MPXV (GenBank: ON563414.3) and VACV WR (Uniprot, Taxon ID 10254) protein sequences via BLAST.

Title: Supplementary data 2: Transcriptomics of HFF cells infected with MPXV

Description: Statistical analysis of transcriptomic changes in HFF cells 6, 12 and 24 hours post MPXV infection (n=4 independent experiments). The two-sided p-values were calculated by Wald test and FDR-adjusted as per standard DESeq2 workflow (see materials and methods).

Title: Supplementary data 3: Total proteome of HFF cells infected with MPXV

Description: Statistical analysis of proteomic changes in HFF cells 6, 12 and 24 hours post MPXV infection (n=5 independent experiments) compared to the respective mock infection. The two-sided p-values were derived from the Bayesian linear model (see materials and methods); no multiple hypothesis correction was performed. The direction of significant proteomic change in VACV (Soday et al., 2019) and MVA (Albarnaz et al., 2022, preprint) infection of HFFF defined by the respective manuscripts was also included.

Title: Supplementary data 4: Phosphoproteome of HFF cells infected with MPXV

Description: Statistical analysis of phosphorylation site changes in HFF cells 6, 12, and 24 hours post MPXV infection (n=5 independent experiments) compared to the respective mock infection. The two-sided p-values were derived from the Bayesian linear model (see materials and methods); no multiple hypothesis correction was performed.

Title: Supplementary data 5: Viral protein and phosphosite kinetics

Description: Kinetics classifications of virus proteins and phosphosites according to their abundances in time-resolved proteomics or phosphoproteomics data of MPXV infection of HFFs.

Title: Supplementary data 6: Biological functions and pathways enriched in HFF cells infected with MPXV

Description: Global enrichment analysis of biological functions and pathways, transcription factors, upstream regulators and kinases based on the changes in the transcriptome, total proteome and phosphoproteome of HFF cells infected with MPXV. The one-sided p-values were calculated by Fisher's Exact test (see materials and methods).

Title: Supplementary data 7: Literature intersection

Description: Intersection of our proteomic and phosphoproteomic data with previous studies.

Title: Supplementary data 8: Results of the network diffusion-based drug and drug target prediction

Description: Results of the network diffusion-based drug and drug target prediction from transcriptome, total proteome and phosphoproteome of HFF cells infected with MPXV. The one-sided p-values were calculated numerically (see materials and methods); no multiple hypothesis correction was performed.

Title: Supplementary data 9: Biological functions and pathways enriched in network diffusion-based drug target prediction

Description: Global enrichment analysis of biological functions and pathways in the network diffusion-based drug target prediction from transcriptome, total proteome and phosphoproteome of HFF cells infected with MPXV. The one-sided p-values were calculated by Fisher's Exact test (see materials and methods); no multiple hypothesis correction was performed.

Title: Supplementary data 10: Viral inhibitor assays

Description: This table contains raw values obtained during the viral inhibitor assay.
