# Supplementary material for: Multi-omics characterization of the monkeypox virus infection
Source: Nat Commun. 2024 Aug 8;15:6778. doi: 10.1038/s41467-024-51074-6 (PMC11310467; doi:10.1038/s41467-024-51074-6)
Supplement: Supplementary file 14 — Reporting Summary [file 41467_2024_51074_MOESM14_ESM.pdf]

nature portfolio

Corresponding author(s): Prof. Andreas Pichlmair

Last updated by author(s): Jul 4, 2024

Reporting Summary

Nature Portfolio wishes to improve the reproducibility of the work that we publish. This form provides structure for consistency and transparency in reporting. For further information on Nature Portfolio policies, see our Editorial Policies and the Editorial Policy Checklist.

Statistics

For all statistical analyses, confirm that the following items are present in the figure legend, table legend, main text, or Methods section.

n/a	Confirmed	
☐	☒	The exact sample size (*n*) for each experimental group/condition, given as a discrete number and unit of measurement
☐	☒	A statement on whether measurements were taken from distinct samples or whether the same sample was measured repeatedly
☐	☒	The statistical test(s) used AND whether they are one- or two-sided<br>*Only common tests should be described solely by name; describe more complex techniques in the Methods section.*
☐	☒	A description of all covariates tested
☐	☒	A description of any assumptions or corrections, such as tests of normality and adjustment for multiple comparisons
☐	☒	A full description of the statistical parameters including central tendency (e.g. means) or other basic estimates (e.g. regression coefficient) AND variation (e.g. standard deviation) or associated estimates of uncertainty (e.g. confidence intervals)
☐	☒	For null hypothesis testing, the test statistic (e.g. *F*, *t*, *r*) with confidence intervals, effect sizes, degrees of freedom and *P* value noted<br>*Give P values as exact values whenever suitable.*
☐	☒	For Bayesian analysis, information on the choice of priors and Markov chain Monte Carlo settings
☐	☒	For hierarchical and complex designs, identification of the appropriate level for tests and full reporting of outcomes
☐	☒	Estimates of effect sizes (e.g. Cohen's *d*, Pearson's *r*), indicating how they were calculated

Our web collection on statistics for biologists contains articles on many of the points above.

Software and code

Policy information about availability of computer code

Data collection	Peptide identification and quantification: MaxQuant (v.2.0.3.1; https://maxquant.net/maxquant/)<br>Imaging data: IncuCyte software (v2020C rev1, Sartorius; commercial)<br>Sequencing data: Dropseq (v1.12, http://mccarrolllab.org/dropseq/)
Data analysis	Bioinformatic analysis was done with R (version 4.1), Julia (version 1.6) and Python (version 3.10) with a set of in-house scripts (https://doi.org/10.5281/zenodo.7757309, https://doi.org/10.5281/zenodo.7752673).<br>The MaxQuant output files were imported into R with the in-house msimportr R package (https://doi.org/10.5281/zenodo.7746897) and analyzed with the msglm package (https://doi.org/10.5281/zenodo.7752068) apart from one modification: the instrumental error of MS intensities was modeled with a mixture of Gaussian and Cauchy instead of Laplacian distribution. The MS1 intensities of protein group/PTM-specific LC peaks (evidence.txt table of MaxQuant output) were used for modeling the protein group or PTM intensities.<br>Transcriptomic data normalization, differential expression analysis and p-value adjustment were performed by the DESeq2 package (version 1.34.0).<br>For gene set enrichment analysis, we used the in-house Julia package OptEnrichedSetCover.jl (https://doi.org/10.5281/zenodo.4536596).<br>For upstream promoter analysis regulated transcripts were submitted to ChEA3 web-based application (https://maayanlab.cloud/chea3/).<br>We performed uniform manifold approximation and projection (UMAP) dimensionality reduction in R (4.0.2) using R package UMAP104 (0.2.6.0) and manually annotated thus obtained clusters.<br>The prediction of host kinase motifs on the host and viral proteins was performed by using the Kinase Library toolbox (kinase-library.phosphosite.org).<br>In silico prediction of protein structure was performed using Alphafold (version 2.3.1) and visualized with Chimera (version 1.3). Electrostatic surface potential of the modeled structure of MPXV H5 dimer was calculated by using the PyMOL plugin APBS electrostatics. Molecular

graphics depictions were produced with the PyMOL software.

In-house R and Julia packages and scripts used for the bioinformatics analysis of the data have been deposited to public GitHub repositories:
msglm: https://doi.org/10.5281/zenodo.7752068
msimportr: https://doi.org/10.5281/zenodo.7746897
OptEnrichSetCover: https://doi.org/10.5281/zenodo.4536596
analysis_utils_jl(package dependencies for the julia packages and scripts used in this manuscript): https://doi.org/10.5281/zenodo.7752673
General scripts: https://doi.org/10.5281/zenodo.7757309

For manuscripts utilizing custom algorithms or software that are central to the research but not yet described in published literature, software must be made available to editors and reviewers. We strongly encourage code deposition in a community repository (e.g. GitHub). See the Nature Portfolio guidelines for submitting code & software for further information.

Data

Policy information about availability of data

All manuscripts must include a data availability statement. This statement should provide the following information, where applicable:

- Accession codes, unique identifiers, or web links for publicly available datasets
- A description of any restrictions on data availability
- For clinical datasets or third party data, please ensure that the statement adheres to our policy

The raw sequencing data for this study have been deposited with the ENA at EMBL-EBI under accession number PRJEB60728. The files of the proteomic datasets and Maxquant output have been deposited to the ProteomeXchange Consortium (http://proteomecentral.proteomexchange.org) via the PRIDE partner repository. This includes the following datasets: Full Proteome HFF/MPXV (PXD040811), Phosphoproteome HFF/MPXV (PXD040889).

Research involving human participants, their data, or biological material

Policy information about studies with human participants or human data. See also policy information about sex, gender (identity/presentation), and sexual orientation and race, ethnicity and racism.

Reporting on sex and gender	*Use the terms sex (biological attribute) and gender (shaped by social and cultural circumstances) carefully in order to avoid confusing both terms. Indicate if findings apply to only one sex or gender; describe whether sex and gender were considered in study design; whether sex and/or gender was determined based on self-reporting or assigned and methods used. Provide in the source data disaggregated sex and gender data, where this information has been collected, and if consent has been obtained for sharing of individual-level data; provide overall numbers in this Reporting Summary. Please state if this information has not been collected. Report sex- and gender-based analyses where performed, justify reasons for lack of sex- and gender-based analysis.*
Reporting on race, ethnicity, or other socially relevant groupings	*Please specify the socially constructed or socially relevant categorization variable(s) used in your manuscript and explain why they were used. Please note that such variables should not be used as proxies for other socially constructed/relevant variables (for example, race or ethnicity should not be used as a proxy for socioeconomic status). Provide clear definitions of the relevant terms used, how they were provided (by the participants/respondents, the researchers, or third parties), and the method(s) used to classify people into the different categories (e.g. self-report, census or administrative data, social media data, etc.) Please provide details about how you controlled for confounding variables in your analyses.*
Population characteristics	*Describe the covariate-relevant population characteristics of the human research participants (e.g. age, genotypic information, past and current diagnosis and treatment categories). If you filled out the behavioural & social sciences study design questions and have nothing to add here, write "See above."*
Recruitment	*Describe how participants were recruited. Outline any potential self-selection bias or other biases that may be present and how these are likely to impact results.*
Ethics oversight	*Identify the organization(s) that approved the study protocol.*

Note that full information on the approval of the study protocol must also be provided in the manuscript.

Field-specific reporting

Please select the one below that is the best fit for your research. If you are not sure, read the appropriate sections before making your selection.

[x] Life sciences [ ] Behavioural & social sciences [ ] Ecological, evolutionary & environmental sciences

For a reference copy of the document with all sections, see nature.com/documents/nr-reporting-summary-flat.pdf

Life sciences study design

All studies must disclose on these points even when the disclosure is negative.

Sample size	The sample sizes were chosen from past knowledge on the good sample size to ensure adequate power. Sample sizes are always indicated in figure legends or related "Methods" section.
Data exclusions	No data were excluded
Replication	For Mass spectrometry, in vitro viral replication experiments, WB analysis, reporter assay and other bioassays, a minimum of three biological experiments were performed independently. All replications were successful.
Randomization	N/A. No randomization was used given the small number of samples and the lack of influence of randomization on the experimental design and experimental approach used. (no animal experiments were performed in this study).
Blinding	N/A. Investigators were not blinded to experimental groups (in vitro experiments required prior knowledge for data interpretation).

Reporting for specific materials, systems and methods

We require information from authors about some types of materials, experimental systems and methods used in many studies. Here, indicate whether each material, system or method listed is relevant to your study. If you are not sure if a list item applies to your research, read the appropriate section before selecting a response.

Materials & experimental systems

n/a	Involved in the study	
☐	☒	Antibodies
☐	☒	Eukaryotic cell lines
☒	☐	Palaeontology and archaeology
☒	☐	Animals and other organisms
☒	☐	Clinical data
☒	☐	Dual use research of concern
☒	☐	Plants

Methods

n/a	Involved in the study	
☒	☐	ChIP-seq
☐	☒	Flow cytometry
☒	☐	MRI-based neuroimaging

Antibodies

Antibodies used	For protein abundance detection via western blot, antibodies against MPXV C19 (Cop-F13, a gift from Michael Way, Francis Crick Institute, 1:8000), MMP14 (Abcam, ab51074, 1:2000), PTGS2 (Cell Signaling, 12282, 1:1000), LMNA (Abcam, ab26300, 1:500), THBS1 (Invitrogen, PA5-85678, 1:1000), DAB2 (Cell Signaling, 12906, 1:1000), CTNNB1 (Sigma-Aldrich, C7207, 1:1000), pCTNNB1-S552 (Cell Signaling, 9566, 1:1000), P38 MAPK (Cell Signaling, 8690, 1:1000), pP38 MAPK - T180/Y182 (Cell Signaling, 4511, 1:1000), HA (coupled to horseradish peroxidase (HRP), Sigma-Aldrich, H6533, 1:2500), StrepTag™ II (coupled to HRP, Sigma-Aldrich, 71591, 1:4000) and ACTB (β-actin) (coupled to HRP, Santa Cruz, sc-47778, 1:2500) were used. Secondary antibodies against mouse (Cell Signaling, 7076, 1:1000) and rabbit (Dako, P0448, 1:2500) IgG were coupled to HRP. For affinity purification–western blotting applications, Streptactin II beads (IBA Lifesciences) were used.<br>For flow cytometry, anti-pan-HLA-APC (clone W6/32, Biolegend, 1:500), anti-ITGB1-FITC (clone Ha2/5, BD Pharmingen, 1:500), anti-MPXV B6 (originally anti-VACV B5, a gift from Michael Way, Francis Crick Institute, 1:1000) and donkey-anti-rat-AF488 (ThermoFisher, A21208, 1:1000) were used.
Validation	Antibodies were validated by infection experiments in this study. Additionally, antibodies have been validated by the manufacturers.

Eukaryotic cell lines

Policy information about cell lines and Sex and Gender in Research

Cell line source(s)	HEK293T (CRL-11268), Vero E6 (CRL-1586), and BSC40 (CRL-2761) cells were purchased from ATCC. HFF-1 (SCRC-1041) and hTERT-BJ1 cells were a kind gift from Prof. Melanie Brinkmann (HZI, Braunschweig, Germany).
Authentication	The identity of all the immortalized cell lines used in this study was confirmed by STR-profiling (Eurofins Medigenomix). Official certification can be provided upon request.
Mycoplasma contamination	All cell lines were tested to be mycoplasma free by standard PCR-based assay.
Commonly misidentified lines (See ICLAC register)	No commonly misidentified cell line was used in this study.

Flow Cytometry

Plots

Confirm that:

- [x] The axis labels state the marker and fluorochrome used (e.g. CD4-FITC).
- [x] The axis scales are clearly visible. Include numbers along axes only for bottom left plot of group (a 'group' is an analysis of identical markers).
- [x] All plots are contour plots with outliers or pseudocolor plots.
- [x] A numerical value for number of cells or percentage (with statistics) is provided.

Methodology

Sample preparation	For each of the three replicates, 1 Mio of HFF cells were infected with MPXV (MOI 3) or left uninfected for 24 hours. The cells were detached by 5 mM EDTA for subsequent staining.
Instrument	Samples were measured on a CytoFlexS flow cytometer (Beckmann Coulter, USA)
Software	FlowJo software (v10.9.0, Tree Star, USA)
Cell population abundance	The cells were not pre-sorted before the analysis.
Gating strategy	*Describe the gating strategy used for all relevant experiments, specifying the preliminary FSC/SSC gates of the starting cell population, indicating where boundaries between "positive" and "negative" staining cell populations are defined.*

- [x] Tick this box to confirm that a figure exemplifying the gating strategy is provided in the Supplementary Information.
